# Piperazine-1,4-diium (*R*)-2-[4-(1-car­boxy­l­atometh­oxy)phen­oxy]propano­ate

**DOI:** 10.1107/S1600536812027213

**Published:** 2012-06-20

**Authors:** Han-Tao Ye, Chang-Yue Ren, Jin-Sheng Gao

**Affiliations:** a Engineering Research Center of Pesticides of Heilongjiang University, Heilongjiang University, Harbin 150050, People’s Republic of China

## Abstract

In the anion of the title mol­ecular salt, C_4_H_12_N_2_^2+^·C_11_H_10_O_6_^2−^, the two acetate groups form torsion angles of 74.1 (1) and 7.1 (1)° with the central benzene ring, and the cation exhibits a chair conformation. In the crystal, N—H⋯O hydrogen bonds link the components into a two-dimensional supra­molecular network lying parallel to the *ab* plane. A number of C—H⋯O inter­actions consolidate the packing.

## Related literature

For the synthesis of the anion, see: Bezwada (2007 ▶). For a similar crystal structure containing the same chiral anion, see: Ren *et al.* (2012 ▶).
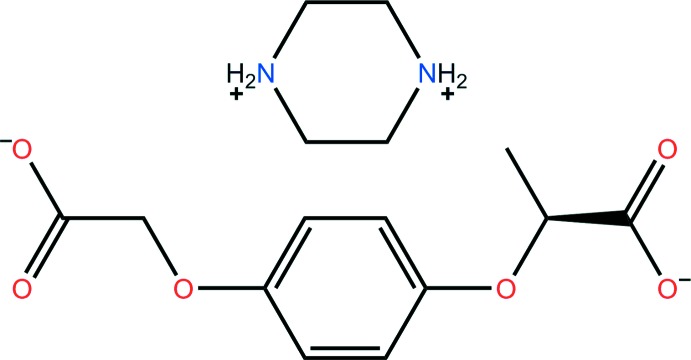


## Experimental

### 

#### Crystal data

C_4_H_12_N_2_^2+^·C_11_H_10_O_6_^2−^
*M* *_r_* = 326.35
Monoclinic, 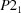

*a* = 6.1210 (12) Å
*b* = 18.134 (4) Å
*c* = 7.0006 (14) Å
β = 90.22 (3)°
*V* = 777.1 (3) Å^3^
*Z* = 2
Mo *K*α radiation
μ = 0.11 mm^−1^
*T* = 293 K
0.56 × 0.22 × 0.17 mm

#### Data collection

Rigaku R-AXIS RAPID diffractometer
Absorption correction: multi-scan (*ABSCOR*; Higashi, 1995 ▶) *T*_min_ = 0.941, *T*_max_ = 0.982
7573 measured reflections
1820 independent reflections
1712 reflections with *I* > 2σ(*I*)
*R*_int_ = 0.029

#### Refinement

*R*[*F* ^2^ > 2σ(*F* ^2^)] = 0.029
*wR*(*F* ^2^) = 0.074
*S* = 1.07
1820 reflections
209 parameters
1 restraint
H atoms treated by a mixture of independent and constrained refinement
Δρ_max_ = 0.16 e Å^−3^
Δρ_min_ = −0.14 e Å^−3^

### 

Data collection: *RAPID-AUTO* (Rigaku, 1998 ▶); cell refinement: *RAPID-AUTO*; data reduction: *CrystalClear* (Rigaku/MSC, 2002 ▶); program(s) used to solve structure: *SHELXS97* (Sheldrick, 2008 ▶); program(s) used to refine structure: *SHELXL97* (Sheldrick, 2008 ▶); molecular graphics: *DIAMOND* (Brandenburg, 1999 ▶; software used to prepare material for publication: *SHELXL97*.

## Supplementary Material

Crystal structure: contains datablock(s) I, global. DOI: 10.1107/S1600536812027213/hb6827sup1.cif

Structure factors: contains datablock(s) I. DOI: 10.1107/S1600536812027213/hb6827Isup2.hkl

Supplementary material file. DOI: 10.1107/S1600536812027213/hb6827Isup3.cml

Additional supplementary materials:  crystallographic information; 3D view; checkCIF report

## Figures and Tables

**Table 1:** Hydrogen-bond geometry (Å, °)

*D*—H⋯*A*	*D*—H	H⋯*A*	*D*⋯*A*	*D*—H⋯*A*
N1—H1*A*⋯O2	0.90	1.89	2.773 (2)	167
N1—H1*B*⋯O1^i^	0.90	1.82	2.681 (2)	160
N2—H2*B*⋯O5^ii^	0.90	1.93	2.803 (2)	163
N2—H2*A*⋯O6^iii^	0.90	1.85	2.711 (2)	159
C9—H9*A*⋯O2^iv^	0.96	2.56	3.419 (3)	150
C12—H12*B*⋯O6^ii^	0.97	2.49	3.339 (2)	146
C13—H13*A*⋯O2^v^	0.97	2.51	3.216 (2)	130
C14—H14*A*⋯O1	0.97	2.58	3.429 (3)	147
C15—H15*A*⋯O6^ii^	0.97	2.54	3.371 (3)	144

Symmetry codes: (i) 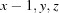; (ii) 
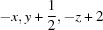
; (iii) 
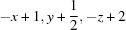
; (iv) 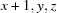; (v) 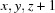.

**Fig. 1.**
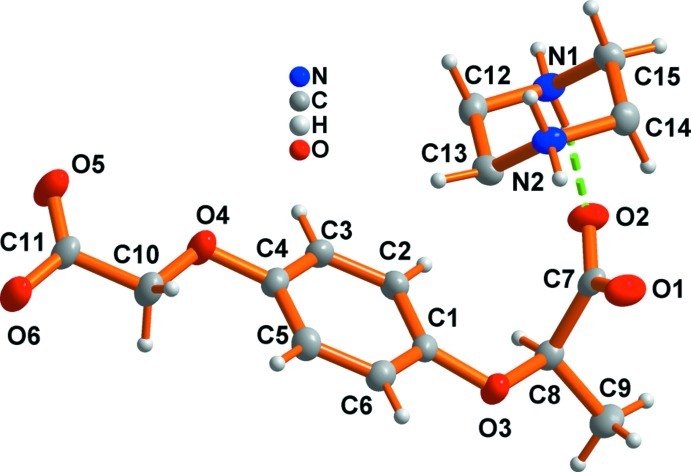
The molecular structure of the title compound, showing displacement ellipsoids at the 50% probability level for non-H atoms.

**Fig. 2.**
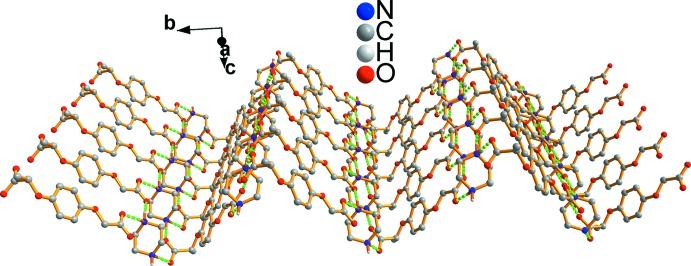
A partial packing view, showing 2D layer structure parallel to the ab plane.

## supplementary crystallographic information

### Comment

Hydrogen bonding is the molecular interaction but strong enough and directional, predictability. Recently, chiral ligands become one of the focus in supramolecular research for their wide applications in catalytic and pharmaceutical industry. However, report about chiral carboxylic acid is few (Ren *et al.* 2012). Herein, we report the synthesis and structure of a new chiral aromatic carboxylic acid contained compound.

The asymmertric unit of title compound, [C_11_H_10_O_6_]^.^[C_4_H_10_N_2_], contains one (*R*)-2-(4-(1-carboxyethoxy)phenoxy)acetate anion and one piperazine-1,4-diium cation (Fig. 1). One acetate group of the anion twist towards a side of the benzenyl plane with the torsion angles of 74.1 (1) °, while the other is almost conplaner with the benzenyl plane with the torsion angles of 7.1 (1) °. In the crystal, a layer structure parallel to the *ab* plane is built up by N—H···O hydrogen bonds linking the anions and cations (Fig. 2, Table 1).

### Experimental

(*R*)-2-(4-(carboxymethoxy)phenoxy)propanoic acid was prepared by the reaction of R-(+)-2-(4-hydroxy-phenoxy)propionic acid and methyl chloroacetate under alkaline condition (Bezwada, 2007). (*R*)-2-(4-(carboxymethoxy)phenoxy)propanoic acid (0.120 g, 0.5 mmol) and 1,4-diazacyclohexane (0.086 g, 1.0 mmol) were dissolved in ethanol (15 ml). After stirring and filtering, colorless block crystals of title compound were obtained upon slow evaporation of the solvent.

### Refinement

H atoms bound to C atoms were placed in calculated positions and treated as riding on their parent atoms, with C—H = 0.93 – 0.98 Å, and with *U*_iso_(H) = 1.2 / 1.5*U*_eq_(C). The N-bond H atoms were located in a difference Fourier map and refined with the N—H bond distance fixed at 0.90 Å. As no signifigcant anomalous scatterings, Friedel pairs were merged, the enantiomer has been assigned by reference to an unchanging chiral centre in the synthetic procedure.

### Crystal data

**Table:**

C_4_H_12_N_2_^+^·C_11_H_10_O_6_^−^	*F*(000) = 348
*M**_r_* = 326.35	*D*_x_ = 1.395 Mg m^−^^3^
Monoclinic, *P*2_1_	Mo *K*α radiation, λ = 0.71073 Å
Hall symbol: P 2yb	Cell parameters from 7107 reflections
*a* = 6.1210 (12) Å	θ = 3.1–27.7°
*b* = 18.134 (4) Å	µ = 0.11 mm^−^^1^
*c* = 7.0006 (14) Å	*T* = 293 K
β = 90.22 (3)°	Block, colorless
*V* = 777.1 (3) Å^3^	0.56 × 0.22 × 0.17 mm
*Z* = 2	

### Data collection

**Table:**

Rigaku R-AXIS RAPID diffractometer	1820 independent reflections
Radiation source: fine-focus sealed tube	1712 reflections with *I* > 2σ(*I*)
Graphite monochromator	*R*_int_ = 0.029
ω scan	θ_max_ = 27.5°, θ_min_ = 3.1°
Absorption correction: multi-scan (*ABSCOR*; Higashi, 1995)	*h* = −7→7
*T*_min_ = 0.941, *T*_max_ = 0.982	*k* = −23→23
7573 measured reflections	*l* = −9→9

### Refinement

**Table:**

Refinement on *F*^2^	Primary atom site location: structure-invariant direct methods
Least-squares matrix: full	Secondary atom site location: difference Fourier map
*R*[*F*^2^ > 2σ(*F*^2^)] = 0.029	Hydrogen site location: inferred from neighbouring sites
*wR*(*F*^2^) = 0.074	H atoms treated by a mixture of independent and constrained refinement
*S* = 1.07	*w* = 1/[σ^2^(*F*_o_^2^) + (0.0448*P*)^2^ + 0.044*P*] where *P* = (*F*_o_^2^ + 2*F*_c_^2^)/3
1820 reflections	(Δ/σ)_max_ < 0.001
209 parameters	Δρ_max_ = 0.16 e Å^−^^3^
1 restraint	Δρ_min_ = −0.14 e Å^−^^3^

### Special details

**Table:**

Geometry. All esds (except the esd in the dihedral angle between two l.s. planes) are estimated using the full covariance matrix. The cell esds are taken into account individually in the estimation of esds in distances, angles and torsion angles; correlations between esds in cell parameters are only used when they are defined by crystal symmetry. An approximate (isotropic) treatment of cell esds is used for estimating esds involving l.s. planes.
Refinement. Refinement of F^2^ against ALL reflections. The weighted R-factor wR and goodness of fit S are based on F^2^, conventional R-factors R are based on F, with F set to zero for negative F^2^. The threshold expression of F^2^ > 2sigma(F^2^) is used only for calculating R-factors(gt) etc. and is not relevant to the choice of reflections for refinement. R-factors based on F^2^ are statistically about twice as large as those based on F, and R- factors based on ALL data will be even larger.

### Fractional atomic coordinates and isotropic or equivalent isotropic displacement parameters (Å^2^)

**Table:**

	*x*	*y*	*z*	*U*_iso_*/*U*_eq_	
N1	0.1268 (2)	0.40697 (9)	0.4052 (2)	0.0270 (3)	
C1	0.6299 (3)	0.20125 (9)	0.4334 (3)	0.0263 (4)	
C2	0.4140 (3)	0.18863 (10)	0.3790 (3)	0.0298 (4)	
H2	0.3625	0.2060	0.2622	0.036*	
C3	0.2751 (3)	0.14978 (11)	0.5005 (3)	0.0308 (4)	
H3	0.1318	0.1407	0.4627	0.037*	
C4	0.3471 (3)	0.12455 (10)	0.6762 (3)	0.0283 (4)	
C5	0.5625 (3)	0.13885 (11)	0.7333 (3)	0.0295 (4)	
H5	0.6129	0.1228	0.8516	0.035*	
C6	0.7006 (3)	0.17731 (10)	0.6113 (3)	0.0284 (4)	
H6	0.8432	0.1872	0.6499	0.034*	
C7	0.6063 (3)	0.34381 (10)	0.2006 (2)	0.0248 (3)	
C8	0.7166 (3)	0.27009 (10)	0.1480 (2)	0.0264 (4)	
H8	0.6118	0.2386	0.0800	0.032*	
C9	0.9189 (3)	0.28043 (12)	0.0266 (3)	0.0364 (4)	
H9A	1.0260	0.3080	0.0972	0.055*	
H9B	0.8809	0.3068	−0.0878	0.055*	
H9C	0.9779	0.2331	−0.0066	0.055*	
C10	0.2861 (3)	0.04605 (11)	0.9413 (3)	0.0318 (4)	
H10A	0.3454	0.0805	1.0339	0.038*	
H10B	0.4051	0.0153	0.8967	0.038*	
C11	0.1160 (3)	−0.00256 (11)	1.0390 (2)	0.0288 (4)	
C12	0.1365 (3)	0.35378 (10)	0.5670 (3)	0.0289 (4)	
H12A	0.1258	0.3039	0.5177	0.035*	
H12B	0.0132	0.3621	0.6509	0.035*	
C13	0.3462 (3)	0.36176 (10)	0.6797 (3)	0.0290 (4)	
H13A	0.3432	0.3288	0.7888	0.035*	
H13B	0.4689	0.3479	0.6001	0.035*	
C14	0.3647 (3)	0.49191 (10)	0.5838 (3)	0.0306 (4)	
H14A	0.4868	0.4831	0.4990	0.037*	
H14B	0.3772	0.5419	0.6322	0.037*	
C15	0.1535 (3)	0.48420 (10)	0.4733 (3)	0.0298 (4)	
H15A	0.0316	0.4974	0.5545	0.036*	
H15B	0.1543	0.5175	0.3649	0.036*	
H1A	0.2331	0.3962	0.3212	0.036*	
H1B	−0.0025	0.4023	0.3446	0.036*	
N2	0.3746 (2)	0.43893 (9)	0.7465 (2)	0.0266 (3)	
H2A	0.5044	0.4433	0.8063	0.032*	
H2B	0.2690	0.4499	0.8309	0.032*	
O1	0.7068 (2)	0.38740 (8)	0.3073 (2)	0.0379 (3)	
O2	0.4179 (2)	0.35462 (9)	0.13462 (19)	0.0364 (3)	
O3	0.7876 (2)	0.23362 (8)	0.32024 (19)	0.0330 (3)	
O4	0.1985 (2)	0.08598 (8)	0.7847 (2)	0.0379 (4)	
O5	−0.0801 (2)	0.00199 (10)	0.9952 (2)	0.0435 (4)	
O6	0.1969 (2)	−0.04445 (9)	1.1639 (2)	0.0371 (3)	

### Atomic displacement parameters (Å^2^)

**Table:**

	*U*^11^	*U*^22^	*U*^33^	*U*^12^	*U*^13^	*U*^23^
N1	0.0224 (7)	0.0360 (8)	0.0225 (7)	0.0005 (6)	−0.0015 (6)	−0.0025 (6)
C1	0.0257 (9)	0.0212 (8)	0.0321 (9)	0.0000 (6)	0.0053 (7)	0.0026 (7)
C2	0.0271 (9)	0.0294 (9)	0.0330 (9)	0.0024 (7)	0.0008 (8)	0.0079 (7)
C3	0.0218 (8)	0.0309 (9)	0.0397 (10)	−0.0002 (7)	0.0006 (7)	0.0076 (8)
C4	0.0259 (9)	0.0279 (8)	0.0311 (9)	−0.0014 (7)	0.0061 (7)	0.0018 (7)
C5	0.0325 (9)	0.0305 (9)	0.0256 (8)	−0.0026 (7)	−0.0002 (7)	0.0022 (7)
C6	0.0257 (8)	0.0290 (9)	0.0304 (9)	−0.0046 (7)	0.0004 (7)	−0.0014 (7)
C7	0.0218 (8)	0.0327 (9)	0.0199 (7)	−0.0026 (7)	0.0040 (6)	0.0034 (7)
C8	0.0251 (8)	0.0299 (9)	0.0241 (8)	−0.0031 (7)	0.0011 (7)	−0.0002 (7)
C9	0.0356 (10)	0.0412 (10)	0.0324 (9)	−0.0004 (9)	0.0118 (8)	−0.0019 (8)
C10	0.0288 (9)	0.0374 (10)	0.0291 (9)	−0.0068 (8)	0.0010 (8)	0.0068 (8)
C11	0.0259 (9)	0.0384 (10)	0.0221 (7)	−0.0067 (7)	0.0031 (7)	0.0034 (7)
C12	0.0311 (9)	0.0263 (9)	0.0294 (8)	−0.0040 (7)	0.0028 (7)	−0.0015 (7)
C13	0.0328 (9)	0.0286 (9)	0.0255 (8)	0.0046 (7)	0.0016 (7)	0.0007 (7)
C14	0.0318 (9)	0.0282 (9)	0.0317 (9)	−0.0053 (7)	−0.0009 (8)	−0.0005 (7)
C15	0.0318 (9)	0.0289 (8)	0.0287 (8)	0.0029 (7)	−0.0030 (7)	0.0021 (7)
N2	0.0236 (7)	0.0338 (8)	0.0224 (7)	0.0025 (6)	−0.0023 (6)	−0.0047 (6)
O1	0.0286 (7)	0.0421 (8)	0.0431 (8)	0.0002 (6)	−0.0032 (6)	−0.0138 (6)
O2	0.0243 (6)	0.0516 (9)	0.0332 (7)	0.0050 (6)	−0.0037 (5)	−0.0028 (6)
O3	0.0241 (6)	0.0382 (7)	0.0366 (7)	−0.0022 (5)	0.0033 (5)	0.0122 (6)
O4	0.0264 (7)	0.0482 (9)	0.0390 (7)	−0.0062 (6)	0.0012 (6)	0.0184 (6)
O5	0.0255 (7)	0.0660 (10)	0.0389 (7)	−0.0083 (7)	−0.0014 (6)	0.0202 (7)
O6	0.0294 (7)	0.0485 (8)	0.0335 (7)	−0.0044 (6)	0.0007 (6)	0.0145 (6)

### Geometric parameters (Å, º)

**Table:**

N1—C15	1.488 (2)	C9—H9B	0.9600
N1—C12	1.489 (2)	C9—H9C	0.9600
N1—H1A	0.9004	C10—O4	1.418 (2)
N1—H1B	0.9000	C10—C11	1.528 (2)
C1—O3	1.382 (2)	C10—H10A	0.9700
C1—C6	1.387 (3)	C10—H10B	0.9700
C1—C2	1.393 (3)	C11—O5	1.240 (2)
C2—C3	1.396 (3)	C11—O6	1.259 (2)
C2—H2	0.9300	C12—C13	1.510 (3)
C3—C4	1.383 (3)	C12—H12A	0.9700
C3—H3	0.9300	C12—H12B	0.9700
C4—O4	1.378 (2)	C13—N2	1.486 (2)
C4—C5	1.400 (3)	C13—H13A	0.9700
C5—C6	1.391 (2)	C13—H13B	0.9700
C5—H5	0.9300	C14—N2	1.491 (2)
C6—H6	0.9300	C14—C15	1.510 (3)
C7—O1	1.248 (2)	C14—H14A	0.9700
C7—O2	1.256 (2)	C14—H14B	0.9700
C7—C8	1.543 (3)	C15—H15A	0.9700
C8—O3	1.441 (2)	C15—H15B	0.9700
C8—C9	1.516 (2)	N2—H2A	0.9000
C8—H8	0.9800	N2—H2B	0.9000
C9—H9A	0.9600		
			
C15—N1—C12	111.22 (13)	O4—C10—H10A	109.1
C15—N1—H1A	109.5	C11—C10—H10A	109.1
C12—N1—H1A	109.2	O4—C10—H10B	109.1
C15—N1—H1B	109.5	C11—C10—H10B	109.1
C12—N1—H1B	109.3	H10A—C10—H10B	107.8
H1A—N1—H1B	108.0	O5—C11—O6	126.13 (17)
O3—C1—C6	115.57 (16)	O5—C11—C10	120.76 (16)
O3—C1—C2	125.27 (17)	O6—C11—C10	113.11 (16)
C6—C1—C2	119.09 (16)	N1—C12—C13	111.51 (15)
C1—C2—C3	119.75 (17)	N1—C12—H12A	109.3
C1—C2—H2	120.1	C13—C12—H12A	109.3
C3—C2—H2	120.1	N1—C12—H12B	109.3
C4—C3—C2	121.06 (17)	C13—C12—H12B	109.3
C4—C3—H3	119.5	H12A—C12—H12B	108.0
C2—C3—H3	119.5	N2—C13—C12	110.66 (15)
O4—C4—C3	116.66 (16)	N2—C13—H13A	109.5
O4—C4—C5	124.07 (17)	C12—C13—H13A	109.5
C3—C4—C5	119.27 (16)	N2—C13—H13B	109.5
C6—C5—C4	119.44 (16)	C12—C13—H13B	109.5
C6—C5—H5	120.3	H13A—C13—H13B	108.1
C4—C5—H5	120.3	N2—C14—C15	111.32 (15)
C1—C6—C5	121.35 (17)	N2—C14—H14A	109.4
C1—C6—H6	119.3	C15—C14—H14A	109.4
C5—C6—H6	119.3	N2—C14—H14B	109.4
O1—C7—O2	124.82 (17)	C15—C14—H14B	109.4
O1—C7—C8	118.46 (16)	H14A—C14—H14B	108.0
O2—C7—C8	116.70 (16)	N1—C15—C14	110.13 (15)
O3—C8—C9	106.36 (15)	N1—C15—H15A	109.6
O3—C8—C7	109.18 (14)	C14—C15—H15A	109.6
C9—C8—C7	112.70 (16)	N1—C15—H15B	109.6
O3—C8—H8	109.5	C14—C15—H15B	109.6
C9—C8—H8	109.5	H15A—C15—H15B	108.1
C7—C8—H8	109.5	C13—N2—C14	111.23 (13)
C8—C9—H9A	109.5	C13—N2—H2A	109.4
C8—C9—H9B	109.5	C14—N2—H2A	109.4
H9A—C9—H9B	109.5	C13—N2—H2B	109.4
C8—C9—H9C	109.5	C14—N2—H2B	109.4
H9A—C9—H9C	109.5	H2A—N2—H2B	108.0
H9B—C9—H9C	109.5	C1—O3—C8	117.74 (14)
O4—C10—C11	112.61 (15)	C4—O4—C10	115.88 (15)

### Hydrogen-bond geometry (Å, º)

**Table:**

*D*—H···*A*	*D*—H	H···*A*	*D*···*A*	*D*—H···*A*
N1—H1*A*···O2	0.90	1.89	2.773 (2)	167
N1—H1*B*···O1^i^	0.90	1.82	2.681 (2)	160
N2—H2*B*···O5^ii^	0.90	1.93	2.803 (2)	163
N2—H2*A*···O6^iii^	0.90	1.85	2.711 (2)	159
C9—H9*A*···O2^iv^	0.96	2.56	3.419 (3)	150
C12—H12*B*···O6^ii^	0.97	2.49	3.339 (2)	146
C13—H13*A*···O2^v^	0.97	2.51	3.216 (2)	130
C14—H14*A*···O1	0.97	2.58	3.429 (3)	147
C15—H15*A*···O6^ii^	0.97	2.54	3.371 (3)	144

Symmetry codes: (i) *x*−1, *y*, *z*; (ii) −*x*, *y*+1/2, −*z*+2; (iii) −*x*+1, *y*+1/2, −*z*+2; (iv) *x*+1, *y*, *z*; (v) *x*, *y*, *z*+1.
